# Mammalian cells lack checkpoints for tetraploidy, aberrant centrosome number, and cytokinesis failure

**DOI:** 10.1186/1471-2121-6-6

**Published:** 2005-02-15

**Authors:** Connie Wong, Tim Stearns

**Affiliations:** 1 Department of Biological Sciences, Stanford University, Stanford, CA 94305, USA; 2 Department of Genetics, Stanford University School of Medicine, Stanford, CA 94305, USA

## Abstract

**Background:**

Mammalian cells have been reported to have a p53-dependent tetraploidy checkpoint that blocks cell cycle progression in G1 in response to failure of cell division. In most cases where the tetraploidy checkpoint has been observed cell division was perturbed by anti-cytoskeleton drug treatments. However, other evidence argues against the existence of a tetraploidy checkpoint. Cells that have failed to divide differ from normal cells in having two nuclei, two centrosomes, a decreased surface to volume ratio, and having undergone an abortive cytokinesis. We tested each of these to determine which, if any, cause a G1 cell cycle arrest.

**Results:**

Primary human diploid fibroblasts with intact cell cycle checkpoints were used in all experiments. Synchronized cells exhibited G1 arrest in response to division failure caused by treatment with either cytochalasin or the myosin II inhibitor blebbistatin. The role of tetraploidy, aberrant centrosome number, and increased cell size were tested by cell/cell and cell/cytoplast fusion experiments; none of these conditions resulted in G1 arrest. Instead we found that various drug treatments of the cells resulted in cellular damage, which was the likely cause of the arrest. When cytokinesis was blocked in the absence of damage-inducing drug treatments no G1 arrest was observed.

**Conclusions:**

We show that neither tetraploidy, aberrant centrosome number, cell size, nor failure of cytokinesis lead to G1 arrest, suggesting that there is no tetraploidy checkpoint. Rather, certain standard synchronization treatments cause damage that is the likely cause of G1 arrest. Since tetraploid cells can cycle when created with minimal manipulation, previous reports of a tetraploidy checkpoint can probably be explained by side effects of the drug treatments used to observe them.

## Background

Cell cycle checkpoints preserve genome integrity by monitoring the fidelity of DNA replication and segregation. In mammalian somatic cells, the best-characterized checkpoints are the DNA damage/replication checkpoints and the mitotic spindle checkpoint. The DNA damage/replication checkpoints result in cell cycle arrest if DNA is not fully replicated, or is damaged [1]. The mitotic spindle checkpoint results in cell cycle arrest prior to anaphase if the spindle is not properly assembled [2].

There is also evidence that defects in events relating to cell division itself can result in cell cycle arrest. Lanni and Jacks [3] and Casenghi et al.[4] found that mammalian cells that had adapted to microtubule depolymerization and exited mitosis without undergoing cytokinesis arrested in G1 of the subsequent cell cycle. Kurimura and Hirano [5] and Andreassen et al. [6] reported that inhibition of cytokinesis with the actin-depolymerizing drug cytochalasin resulted in a similar arrest in G1 of the subsequent cell cycle. These treatments resulted in cells that were tetraploid, and Andreassen et al. [6] proposed that the cell cycle arrest was triggered by ploidy, terming this effect a "tetraploidy checkpoint".

Other evidence suggests that mammalian cells are not sensitive to tetraploidy. Rao and Johnson used cell fusion to study the regulation of DNA synthesis and mitosis by fusing cells at different cell cycle stages [7,8]. Binucleate tetraploid cells resulting from fusion between cells in different cell cycle stages were able to progress through the cell cycle. Uetake and Sluder ([9], reviewed in [10]) reported that inhibition of cytokinesis with a low dose of cytochalasin also allowed cell cycle progression. Most strikingly, there are rare cases of human infants born with fully tetraploid karyotypes [11]. Although these individuals have severe defects, their existence argues against tetraploidy as a trigger for cell cycle arrest.

Here we investigate whether tetraploidy or other cellular defects in binucleate cells lead to cell cycle arrest. We show that neither tetraploidy, aberrant centrosome number, cell size, nor failure of cytokinesis lead to G1 arrest, suggesting that there is no tetraploidy checkpoint. Rather, certain standard synchronization treatments cause DNA damage that is the likely cause of G1 arrest.

## Results and discussion

**Figure 1:**
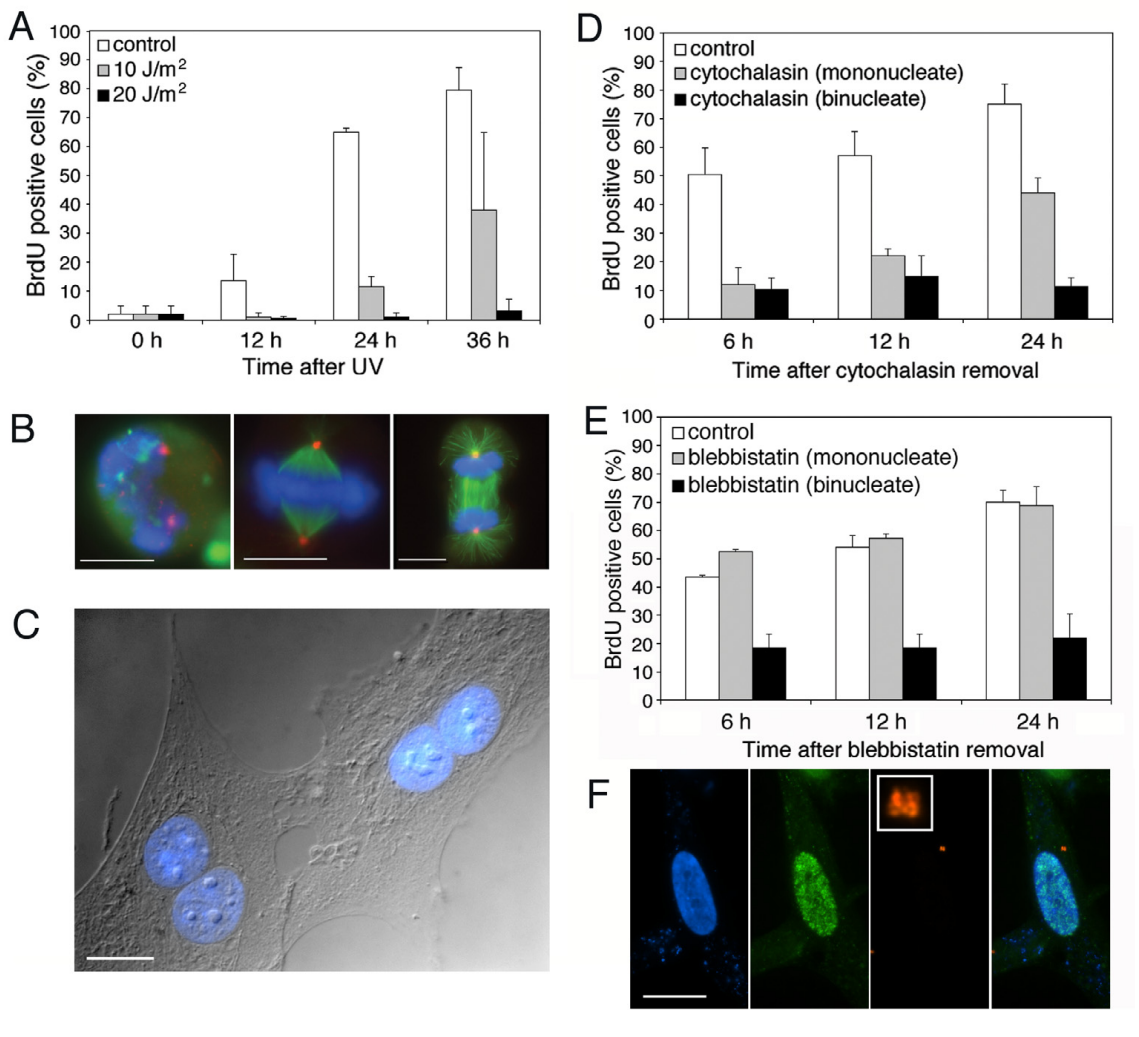
**Cell cycle responses of human diploid fibroblast (HDF) cells. **(A) Response to DNA damage. HDF cells were exposed to 0, 10 or 20 J/m^2 ^ultraviolet light and entry into S phase was assayed by BrdU incorporation. For each bar n ≥ 300 cells. (B) Recovery from nocodazole arrest. HDF cells were arrested in mitosis by double thymidine block followed by nocodazole (left) and released for 30 min. (center and right). DNA, blue; α-tubulin, green; γ-tubulin, red. (C) Example of binucleate cells created by cytochalasin-induced cytokinesis failure. DNA, blue. (D) Cell cycle progression of HDF cells in response to cytokinesis failure induced with 2 μM cytochalasin. Cells were assayed for BrdU incorporation at the indicated times after removal of cytochalasin. "control" cells were not treated with cytochalasin; "cytochalasin (mononucleate)" cells were treated, but completed cytokinesis, and "cytochalasin (binucleate)" cells were treated and failed to divide in cytokinesis. For each bar n ≥ 300 cells. (E) Cell cycle progression of HDF cells in response to cytokinesis failure induced with 12.5 μM blebbistatin. Cells were assayed for BrdU incorporation at the indicated times after removal of blebbistatin. "control" cells were not treated with blebbistatin; "blebbistatin (mononucleate)" cells were treated, but completed cytokinesis, and "blebbistatin (binucleate)" cells were treated and failed to divide in cytokinesis. For each bar n ≥ 300 cells. (F) Cell cycle progression in response to the presence of extra centrosomes. Image shows the product of fusion between a G1 cell and a G1 cytoplast. 24 h after fusion this cell has four centrosomes, indicating that it has undergone centrosome duplication, and has incorporated BrdU, indicating that it has entered S phase. DNA, blue; BrdU, green; pericentrin, red. Punctate blue staining is due to cell surface marker used to identify fusion products [12]. Scale bars represent 10 μm.

Immortalized cell lines often have altered checkpoints, therefore we used early passage primary cells to investigate the tetraploidy checkpoint. All experiments were performed with human diploid fibroblasts (HDF) from infant foreskin and used prior to passage 10. We had previously developed methods for synchronizing these cells [12], and tested them here for the presence of normal checkpoint mechanisms. First, the levels of p53 were determined by western blotting and found to be similar to other p53+/+ cell lines (not shown). Second, we tested for a functional DNA damage response. G1 phase HDF cells were released from serum starvation and irradiated with ultraviolet (UV) light. The cells were then assayed for entry into S phase by 5-bromodeoxyuridine (BrdU) incorporation. The HDF cells exhibited a normal DNA damage response; at a low dose of UV, cells were delayed by about 12 h for entry into S phase, and at a higher dose most cells did not enter S phase even 36 h after irradiation (Figure 1A). Third, we tested for a functional spindle checkpoint. Exponentially-growing HDF cells were treated with nocodazole for 12 h to depolymerize microtubules, and assayed by light microscopy. Nocodazole treatment caused a 6-fold increase in the mitotic index, indicating that the cells had a functional spindle checkpoint.

We first tested HDF cells for the previously described G1 arrest following cytochalasin-induced failure of cytokinesis [6]. Cells were synchronized in mitosis by double thymidine arrest followed by nocodazole treatment (Figure 1B), then released for 30 min, after which most cells had a bipolar spindle. Cells were then allowed to proceed into interphase in medium containing BrdU, +/- 2 μM cytochalasin. By 10 h after the addition of cytochalasin, both control and cytochalasin-treated cells had exited mitosis; approximately 30% of the cytochalasin-treated cells had two nuclei (binucleate) (Figure 1C) and the remainder had a single nucleus (mononucleate), presumably having completed cytokinesis successfully. Thus there were two types of control cells in these experiments: cells that had not experienced the drug, and cells that had experienced the drug, but remained mononucleate. The cultures were washed at this point to remove drug and allowed to proceed in the cell cycle.

At 6 h after the removal of cytochalasin, 50% of the untreated control cells had entered S phase, whereas only about 10% of either the mononucleate or binucleate cytochalasin-treated cells had entered S phase (Figure 1D); these numbers changed only slightly by 12 h. However, at 24 h after cytochalasin removal, 75% of the control cells and 44% of the mononucleate cytochalasin-treated cells had entered S phase, whereas only 11% of the cytochalasin-treated binucleate cells had entered S phase. Similar results were obtained with 5 μM and 10 μM cytochalasin (not shown). Thus, binucleate HDF cells resulting from cytochalasin-induced failure of cytokinesis did arrest in G1, as previously described for other cells [6].

A potential problem with cytochalasin treatment is that depolymerization of the actin cytoskeleton is likely to have effects other than blocking cytokinesis. Indeed, we found that even at 2 μM, cytochalasin had a strong cytotoxic effect, delaying cell cycle progression significantly, with slow recovery after release (data not shown and [13]). To determine whether the effect was specific to cytochalasin, the above experiment was repeated using two other drugs that inhibit cytokinesis: blebbistatin and aurora kinase inhibitor-1 (AKI-1). Blebbistatin is an inhibitor of non-muscle myosin II, the motor protein that provides the force for furrow ingression during cytokinesis [14]. AKI-1 inhibits the aurora family of kinases, which play important roles in mitosis and cytokinesis [15].

HDF cells were synchronized in mitosis by double thymidine block followed by nocodazole treatment, then released into medium containing BrdU, +/- 12.5 μM blebbistatin. By 10 h after the addition of blebbistatin, most cells had exited mitosis; in the presence of blebbistatin approximately 30% of the cells were binucleate and the remaining cells were mononucleate, presumably completing cytokinesis successfully. Blebbistatin was removed, and cells were assayed for S phase entry over time.

At 6 h after the removal of blebbistatin, 44% of the untreated control cells and 53% of the mononucleate blebbistatin-treated cells had entered S phase, whereas only 18% of the binucleate blebbistatin-treated cells had entered S phase (Figure 1E). By 24 h the fraction of both untreated and blebbistatin-treated mononucleate cells that had entered S phase rose to about 70%, whereas the fraction of binucleate cells that had entered S phase remained at about 20% (Figure 1E). Similar results were obtained with 25 μM and 50 μM blebbistatin, as well as with 5 μM AKI-1 (not shown). This indicates that synchronized mitotic cells that failed cytokinesis became arrested in G1 regardless of the specific inhibitor used.

Cells that have failed to divide after mitosis differ from normal cells in that they have two nuclei, two centrosomes, and a decreased surface area to volume ratio. We tested each of these defects individually for an effect on G1 arrest. To test the role of centrosome number, serum-starved G0 cells were fused with enucleated G0 cytoplasts to create cells with two centrosomes, but only one diploid nucleus (Figure 1F). The cell-cytoplast fusions were released from G0 into BrdU-containing medium and allowed to proceed through the cell cycle. The fused cells were compared to cells in the population that had experienced the fusion treatment but had not fused. At 24 h after fusion, 66+/-15% of cytoplast-cell fusions with an extra centrosome had entered S phase, and 63+/-11% of unfused control cells had entered S phase. Therefore the presence of an extra centrosome at G1 does not delay S phase entry and is not responsible for the G1 arrest in binucleate cells resulting from cytochalasin-induced failure of cytokinesis.

To test the role of tetraploidy, serum-starved HDF cells were fused to create binucleate cells. Creating binucleate cells by fusion avoided disruption of the actin cytoskeleton, allowing us to examine the effect of ploidy alone. The binucleate cells resulting from fusion were both tetraploid and had two centrosomes; we showed above that centrosome number was not a factor in the G1 arrest. As above, unfused cells in the population served as an internal control. At 24 h after fusion, 72+/-2% of the unfused cells and 75+/-1% of the fused, binucleate, cells had entered S phase. Therefore, tetraploidy does not cause the observed G1 arrest resulting from cytochalasin-induced failure of cytokinesis.

**Figure 2:**
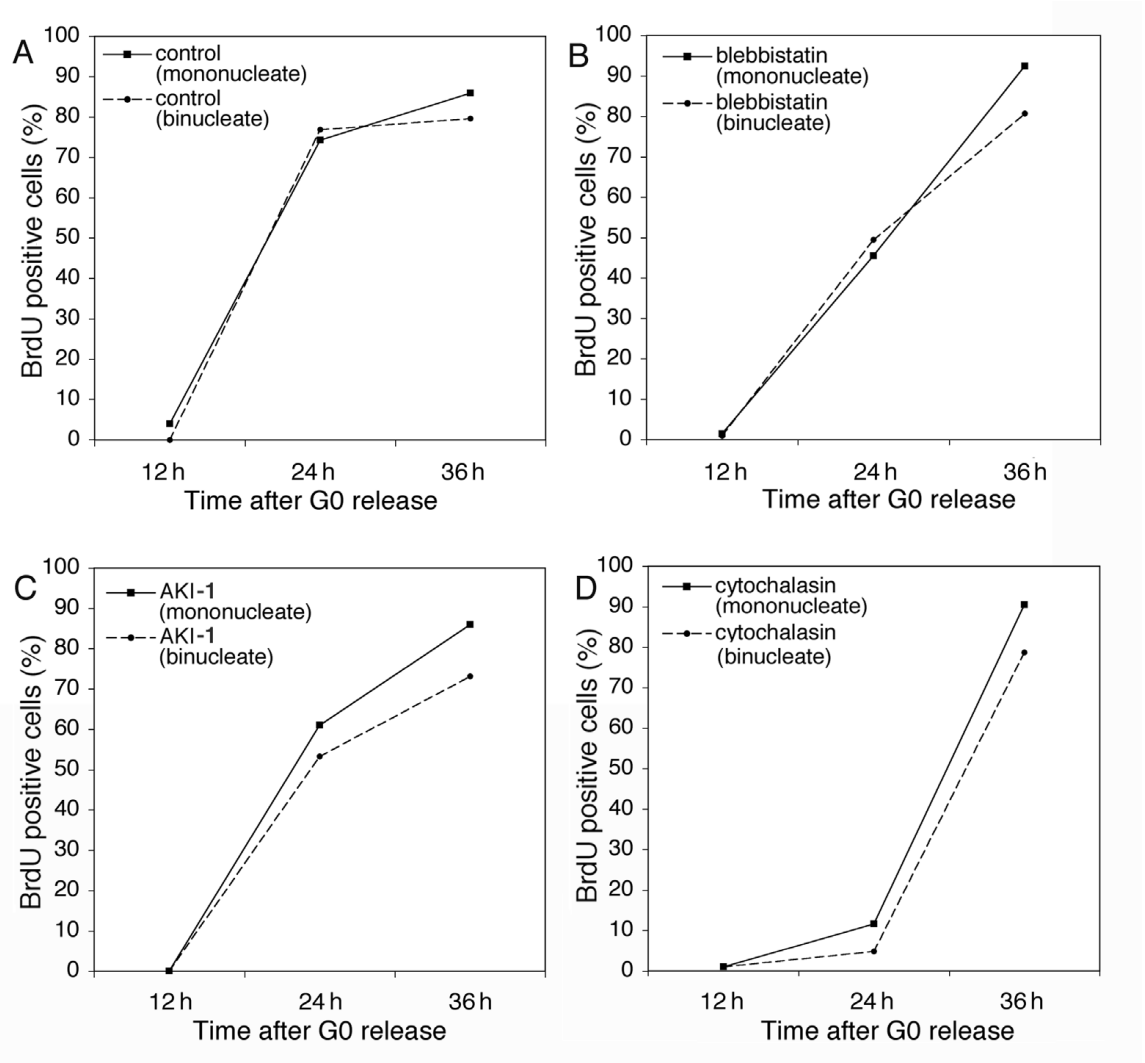
**Cytokinesis inhibitors do not block the G1 to S phase progression of binucleate HDF cells. **Serum-starved G0 cells were fused and released into medium containing BrdU and (A) no drug, (B) 25 μM blebbistatin, (C) 5 μM AKI-1, or (D) 5 μM cytochalasin. Some cells remain unfused after the fusion treatment, and were used as mononucleate controls. Time points were taken to assay for S phase entry.

Cells that fail to divide at cytokinesis are larger than normal cells. Larger cells have a decreased surface area to volume ratio, which might affect the response to perturbation of the cytoskeleton. Thus, the apparent sensitivity to cytokinesis failure might derive directly from a difference in size. To test this, we created large binucleate cells by fusing serum-starved G0 cells to each other. The fusion products were released into growth medium for 3 h to allow for reattachment to the culture substrate. We then added 25 μM blebbistatin, 5 μM cytochalasin, or 5 μM AKI-1 to cells for 10 h, followed by release into BrdU-containing medium. Figure 2 shows that mononucleate and binucleate cells in the control and drug-treated populations entered S phase with similar kinetics. Note that in the cells treated with cytochalasin there was a significant delay in S phase entry, consistent with the cytotoxicity of cytochalasin that we and others have described [13]. These results demonstrate that binucleate cells are not more sensitive to cytokinesis inhibitors due to their increased size.

**Figure 3:**
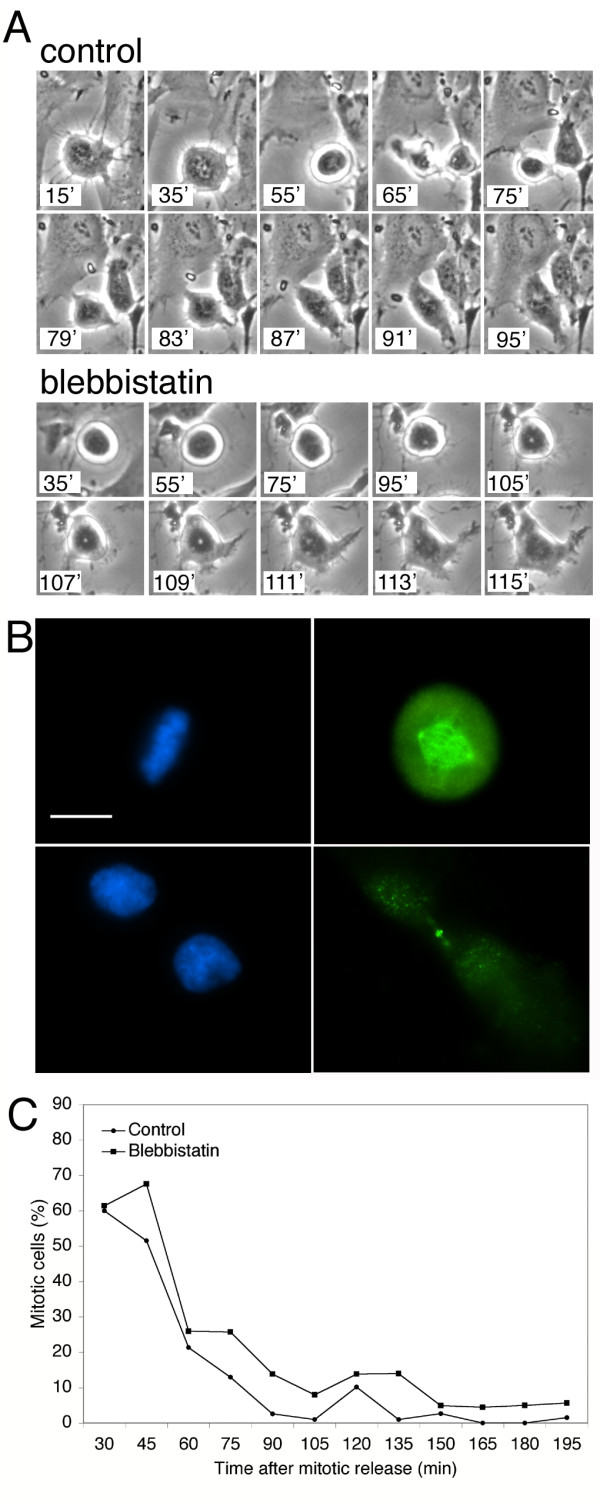
**Cytokinesis failure does not significantly delay the exit frommitosis. **(A) Images from time-lapse series of HDF cells at the indicated times after release from nocodazole-mediated mitotic arrest. "Control" cells were not treated with blebbistatin; "Blebbistatin" cells were treated with blebbistatin beginning at 30 min after release from nocodazole. (B) MPM-2 immunofluorescence as a marker for mitotic exit. Fluorescence image of a mitotic cell with condensed DNA and intense MPM-2 staining (top) and a cytokinetic cell with decondensed DNA and diminished MPM-2 staining (bottom). DNA, blue; MPM-2, green. Scale bar represents 10 μm. (C) Mitotic index of control and blebbistatin-treated cells after mitotic release. "Control" cells were not treated with blebbistatin; "Blebbistatin" cells were treated with blebbistatin beginning at 30 min after release from nocodazole. Mitotic index was determined by MPM-2 staining and DNA morphology, as in (B). For each point n = 100 cells.

If cells were sensitive to failure of cytokinesis, one might expect that the sensitivity would be expressed as a delay in exit from mitosis, a time when a cytokinetic defect could be corrected. This would be similar to the known DNA damage, DNA replication and spindle assembly checkpoints [16]. We tested in two ways whether mammalian cells delay the exit from mitosis in response to cytokinesis failure. First, HDF cells were imaged by time-lapse microscopy as they progressed through mitosis in the presence or absence of blebbistatin. Cells were synchronized in mitosis by nocodazole treatment, then released for 30 min, when 25 μM blebbistatin was added. Control cells (n = 5) exhibited cytokinetic constrictions beginning about 60 min after release from nocodazole. These cells flattened and began to spread, signaling the end of mitosis, about 85 min after release (Figure 3A). Blebbistatin-treated cells (n = 9) did not undergo observable cytokinesis, as expected, but did flatten and spread about 110 min after release from nocodazole (Figure 3A). We also tested for a delay in mitotic exit by staining with MPM-2, and antibody specific for mitotic phosphoepitopes [17]. Cells that were MPM-2 positive and had condensed DNA were considered to be in mitosis (Figure 3B). After release from nocodazole arrest, the fraction of mitotic cells declined in both the control and blebbistatin-treated populations with only a slight delay apparent in the blebbistatin-treated cells (Figure 3C). Both assays showed that blebbistatin treatment resulted in only a brief delay in the exit from mitosis, suggesting that failure of cytokinesis does not trigger a checkpoint-like arrest.

**Figure 4:**
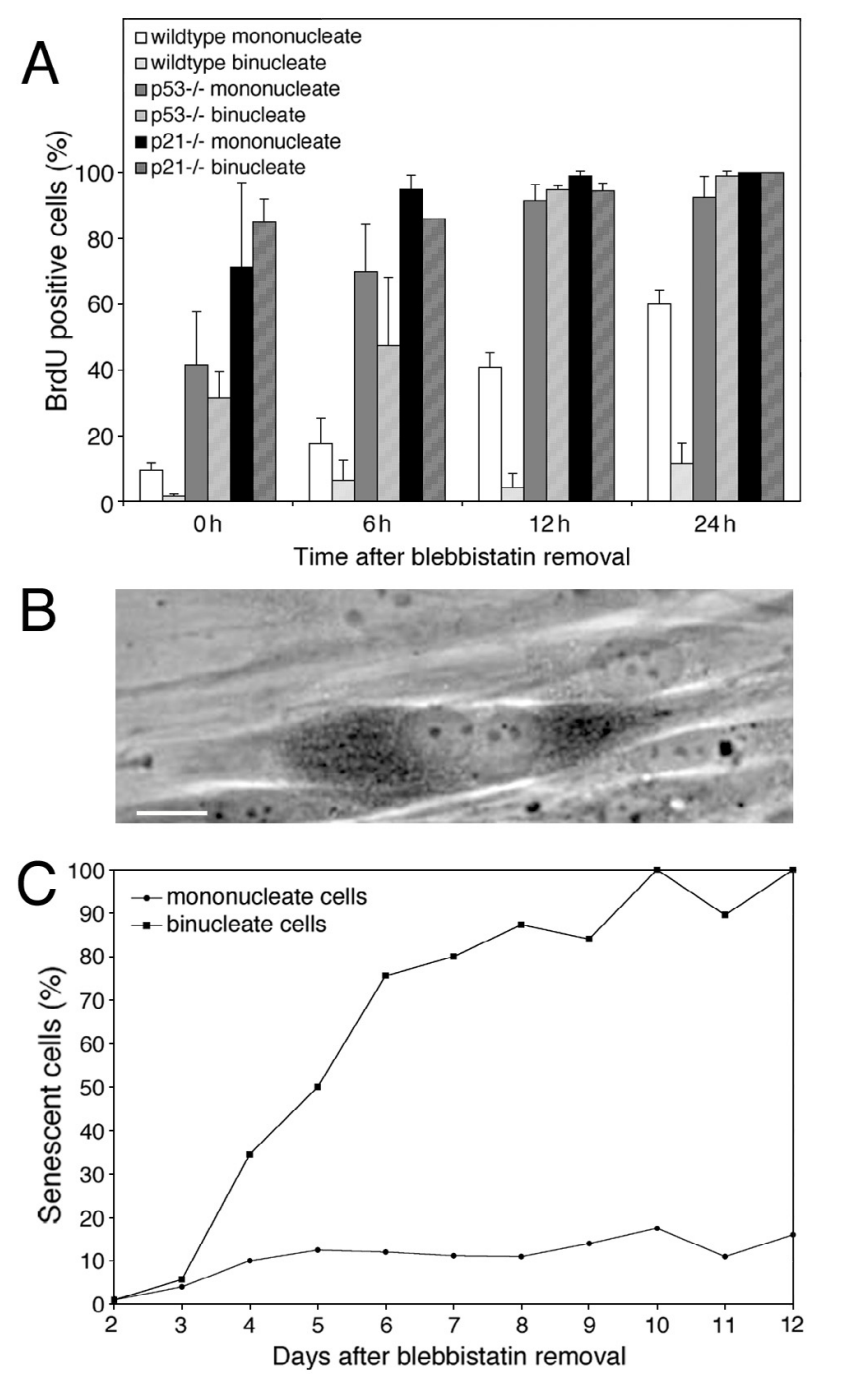
**Cells arrested in G1 by cytokinesis failure enter p53- and p21-dependent premature senescence. **(A) p53 pathway dependence of the G1 arrest following cytokinesis failure. Wt, p53-/- and p21-/- mouse embryo fibroblasts were synchronized and treated with blebbistatin and assayed as in Figure 1E. For each bar n ≥ 200 cells. (B) G1 arrested binucleate cells entered premature senescence as assayed by senescence-associated β-galactosidase activity (SA-β-gal). The dark stain in the binucleate cell shown is the reaction product of Xgal cleavage. Scale bar represents 10 μm. (C) Time course of appearance of senescent cells. Mononucleate and binucleate cells were assayed for SA-β-gal at the indicated days after blebbistatin removal. For each point n = 60–100 cells.

Since we had ruled out most of the cellular defects associated with division failure as being the cause of the G1 arrest, we attempted to further characterize the arrest. Andreassen et al. [6] reported that p53 is important in the G1 arrest caused by cytokinesis failure. To test the role of the p53 pathway, we repeated the blebbistatin experiment above with wt, p53 -/-, and p21 -/- mouse embryonic fibroblast (MEF) cells. Wt MEF cells behaved similarly to the HDF cells, arresting in G1 in response to cytokinesis failure (Figure 4A). However, in p53 -/- and p21 -/- MEF cells both binucleate and mononucleate cells entered S phase with the same kinetics (Figure 4A). Thus, the p53-p21 pathway is required for the G1 arrest of binucleate cells.

p53-p21-dependent G1 arrest often results in either apoptosis or senescence [18,19]. To determine the fate of the G1-arrested products of a failed cytokinesis, binucleate HDF cells were prepared using blebbistatin as described above. Blebbistatin was then removed and the cells were assayed by microscopy. The binucleate cells persisted in the population over the course of more than two weeks, consistent with these cells being permanently arrested in the cell cycle. The binucleate cells did not undergo apoptosis, as assayed by morphology and staining with annexin V, an early marker of apoptosis (not shown). However, the binucleate cells did develop several hallmarks of senescence, including becoming flattened and enlarged, and accumulating senescence-associated β-galactosidase activity (Figure 4B). As the criteria for defining cellular senescence are not firmly established [20], we will refer to this phenotype as "senescent-like". At 4 days after blebbistatin removal approximately 35% of binucleate cells and 10% of mononucleate cells were senescent-like; by 12 days virtually all of the binucleate cells, but only 10% of mononucleate cells, were senescent-like (Figure 4C).

We have shown that failure of division of synchronized cells results in a p53-dependent arrest, but that the arrest is not due to ploidy, centrosome number, or cell size, and that the arrest is not preceded by a delay in mitotic exit, suggesting that it is not a classical checkpoint. The characteristics of the arrest are similar to those of the G1 arrest caused by the DNA damage checkpoint in HDF cells, which respond to irreparable DNA damage by entering senescence, instead of apoptosis [21]. These similarities led us to test whether the binucleate G1 arrest might actually be due to DNA damage suffered during the treatment. Cells were synchronized in mitosis by the double thymidine – nocodazole regimen described above and treated with 25 μM blebbistatin. The binucleate cells were released from blebbistatin for 1 h, 3 days, and 8 days respectively, then stained for γ-H2AX, a marker of DNA damage [22]. As a positive control for DNA damage, asynchronous cells were treated with 1 mM hydrogen peroxide for 30 min, allowed to recover in medium for 1 h, and stained for γ-H2AX.

**Figure 5:**
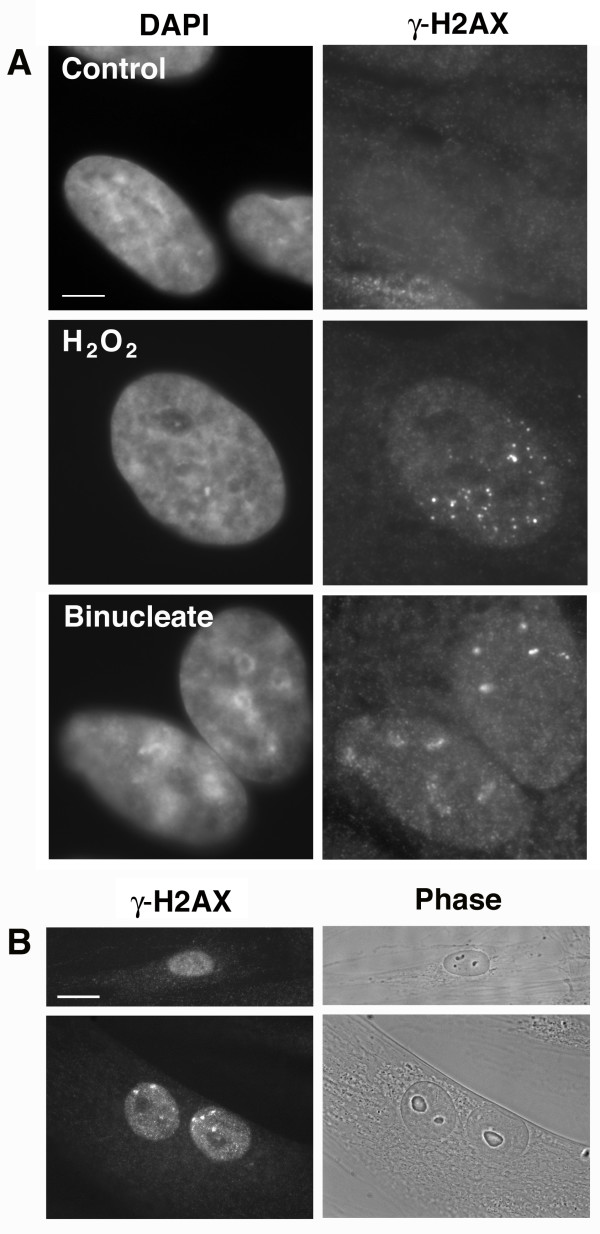
**Binucleate cells contain nuclear γ-H2AX foci. **(A) Binucleate cells were prepared by synchronization and treatment with 25 μM blebbistatin as described above, then stained for the DNA damage marker γ-H2AX. Untreated control cells (top) did not contain any visible γ-H2AX foci, whereas binucleate cells, after released from blebbistatin for 1 h, (bottom) contained γ-H2AX foci that were similar to those of cells treated with H_2_O_2 _(middle). Scale bar represents 2.5 μm. (B) Culture was continued for 8 days after release from blebbistatin. Most mononucleate cells lacked γ-H2AX foci (top), whereas approximately 30% of binucleate cells still contained nuclear γ-H2AX foci (bottom). The binucleate cells were also flattened and enlarged, consistent with a senescent-like arrest. Scale bar represents 10 μm.

In the untreated control cells, only 3.4% of cells contained γ-H2AX foci, whereas in the hydrogen peroxide treated cells, 33% of cells contained γ-H2AX foci (Figure 5A). Remarkably, at 1 h after release of synchronized cells from blebbistatin, 52% of the binucleate cells contained γ-H2AX foci in one or both nuclei (Figure 5A), suggesting the presence of DNA damage. However, we found that 32% of the mononucleate cells that successfully completed cytokinesis after blebbistatin treatment also contained γ-H2AX foci. This suggested that the observed DNA damage might not be the result of division failure per se, and therefore might have occurred prior to the addition of blebbistatin, possibly during cell synchronization. At 3 days after release from blebbistatin, 30% of the binucleate cells and 11% of the mononucleate cells contained visible γ-H2AX foci. At 8 days after release from blebbistatin, 29% of the binucleate cells and only 6% of the mononucleate cells contained γ-H2AX foci. Most of the binucleate cells also displayed senescent-like phenotypes at 8 days after the removal of blebbistatin (Figure 5B).

The binucleate cells that persisted in culture were arrested in G1, as they did not incorporate BrdU after the previous round of mitosis, and they did not proceed to the next round of mitosis, as evidenced by the preservation of the binucleate phenotype. The presence and persistence of nuclear γ-H2AX foci in the G1-arrested binucleate cells suggested that DNA damage might be the cause of the arrest. However, not all the arrested binuclear cells contained visible γ-H2AX foci, indicating that γ-H2AX-associated DNA damage might not be the only cause of the arrest. The percentage of binucleate cells with nuclear γ-H2AX decreased from 52% to 29% over 8 days of culturing, possibly indicating that some cells were able to correct the DNA damage after being arrested in G1 for several days. In contrast, the percentage of mononucleate cells that displayed γ-H2AX foci decreased dramatically over 8 days of culturing, however this was likely due to proliferation of normal mononucleate cells in the culture rather than a difference in response of the mononucleate and binucleate cells to the treatment.

**Figure 6:**
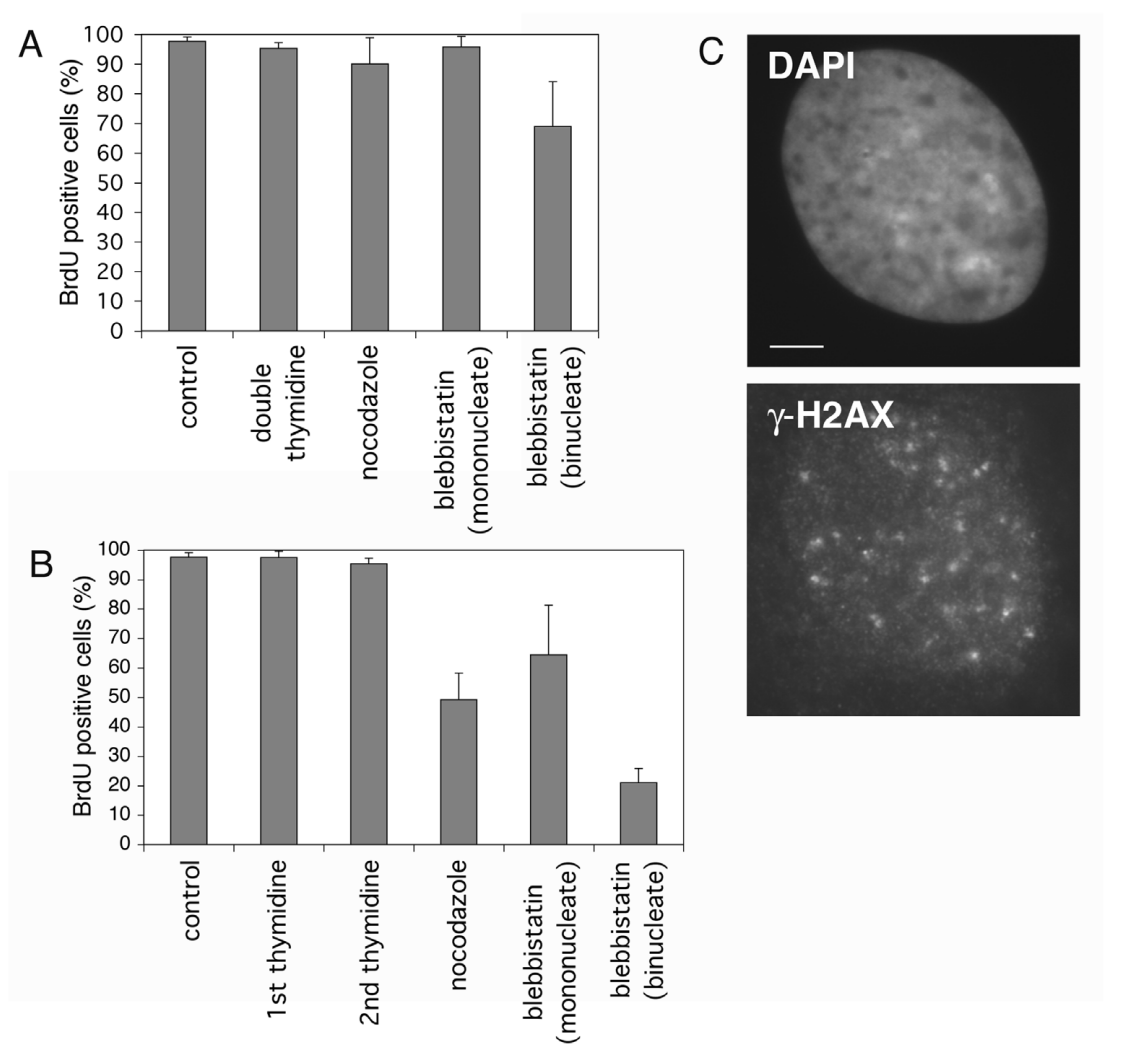
**The combination of double thymidine block and nocodazole treatment causes DNA damage in HDF cells **(A) Asynchronous HDF cells were treated with either double thymidine, nocodazole, or blebbistatin individually, then released into BrdU-containing growth media and assayed for S phase entry. For each bar n ≥ 200 cells. (B) Cells were subjected to the treatments in the order double thymidine, nocodazole, blebbistatin. Samples of cells were taken after release from each drug, and S phase entry was assayed. For each bar n ≥ 200 cells. (C) Cells were treated with double thymidine block followed by nocodazole, then stained for γ-H2AX. Scale bar represents 2.5 μm.

The presence of γ-H2AX foci in the mononucleate cells that successfully completed cytokinesis after blebbistatin treatment suggested that the DNA damage might have been the result of the synchronization treatments, prior to the addition of blebbistatin. Therefore, we tested whether any of the cell synchronization treatments alone had an effect on cell cycle progression. Asynchronous cells were treated with double thymidine block, nocodazole, or blebbistatin individually, following the same protocols used above in multiple treatments. Cells were released from drug and S phase entry was assayed at time points. Figure 6A shows that none of the drug treatments resulted in a substantial failure of cell cycle progression after release. Most importantly, we found that most of the binucleate cells that resulted from cytokinesis failure with blebbistatin treatment alone were able to enter S phase normally after release. This result indicates that there is no cytokinesis checkpoint, in accord with the results of Uetake and Sluder [9].

Since none of the single drug treatments resulted in a cell cycle arrest, we reasoned that some combination of the treatments must be responsible. To determine which combination of treatments caused a G1 arrest, cells were subjected to the treatments in the order double thymidine, nocodazole, blebbistatin. Samples of cells were taken after release from each drug, and S phase entry was assayed (Figure 6B). Although neither double thymidine nor nocodazole arrest and release alone resulted in a G1 arrest, the combination of them did; only about 45% of such cells progressed into S phase. The addition of blebbistatin to the treatment did not cause a further reduction in the fraction of cells entering S phase; about 64% of the mononucleate cells progressed into S phase. However, only about 22% of the binucleate cells that failed cytokinesis after blebbistatin treatment progressed into S phase. This indicated that the G1 arrest in our experiments is due to the double thymidine block, followed by nocodazole treatment, and that binucleate cells are more susceptible to this effect.

To determine whether the thymidine – nocodazole combination caused the DNA damage we observed, cells were treated with both drugs as above and stained for γ-H2AX. At 3 h after release from the drug treatments, about 33% of cells had γ-H2AX foci (Figure 6C). Thus the thymidine – nocodazole synchronization treatment caused the DNA damage that resulted in cells becoming arrested in G1. Given these results we suggest a simple model for the increased susceptibility of binucleate cells: those cells that failed cytokinesis are more likely to become arrested in G1 because they contain two nuclei, and thus have twice the chance of inheriting DNA damage compared with cells that successfully divided.

## Conclusions

We have shown that tetraploidy, aberrant centrosome number, increased cell size, and failure of cytokinesis do not lead to G1 arrest in primary human diploid fibroblasts. Rather, we found that the observed G1 arrest in cells that have failed to divide is likely due to cellular damage caused by standard synchronization treatments. We note that all published observations of a G1 arrest in response to division failure involved extensive manipulation of mammalian cells in culture. It seems likely that these manipulations resulted in DNA damage, or in other damage, that resulted in a G1 arrest, but was not directly associated with division failure. For example, Uetake and Sluder [9] found that supplementing the culture substrate with fibronectin allowed binucleate cells formed by cytochalasin treatment to progress through the cell cycle, suggesting that cell adhesion was defective in the drug-treated cells. Given that binucleate cells clearly can cycle when formed with minimal manipulation, it is likely that all previous reports of a tetraploidy checkpoint can be explained by side effects of the drug treatments used to observe them.

## Methods

### Cell methods

Human diploid fibroblasts (HDFs) were from infant foreskin. Wt, p53 -/- and p21 -/- mouse embryo fibroblasts (MEFs) were the kind gift of Laura Attardi (Stanford, CA). HDFs and MEFs were cultured in DMEM (Gibco) with 10% fetal bovine serum. HDFs were used prior to passage 10 and MEFs were used prior to passage 5. HDFs were synchronized in G0 by serum starvation [12] and S phase by double thymidine block [23], as described. In the cell fusion experiments, serum-starved G0 cells, or cytoplasts derived from those cells by centrifugation, were fused with serum-starved G0 cells, as described [12]. Immunocytochemistry was as described [12]. Live cell imaging was with a Nikon Diaphot microscope equipped with an environmental chamber allowing incubation at 37°. Images were collected with a CCD camera (Photometrics) and processed with Openlab (Improvision) and Photoshop (Adobe) software. Senescence-associated β-galactosidase activity (SA-β-gal) was assayed as described [24].

### Assay for S phase entry by BrdU incorporation

Cells were incubated with 20 μM BrdU (Sigma) for indicated times and fixed in -20°C methanol for at least 10 min. Fixed cells were treated with DNase I (Boehringer Mannheim) and exonuclease III (New England Biolabs) to expose the BrdU epitope prior to incubation with anti-BrdU antibodies, as described [12]. Nuclei were visualized by staining 4',6-diamidino-2-phenylindole (DAPI). Cells were observed with a Zeiss Axioskop microscope with a Zeiss Plan-Neofluar 100/1.3 objective, and images were collected with a cooled-CCD camera (Hamamatsu) controlled by Openlab software.

### Assay for DNA damage by γ-H2AX staining

Cells were fixed with 2% paraformaldehyde at room temperature for 10 min, washed 3× with PBS, then permeabilized with-20°C methanol for 5 min and stained with 27 ng/ml γ-H2AX antibody (Trevigen, MD). Only cells with multiple, clearly labeled foci were counted as being γ-H2AX positive.

### Drug-induced cytokinesis failure

Cells synchronized in S phase by double thymidine block were released from the block for 6 h to allow completion of S phase. Nocodazole (100 ng/ml) (US Biological) was then added for 6 h to arrest the cells in mitosis. Cells were released from the mitotic arrest for 30 min, during which time most cells formed a bipolar mitotic spindle (Fig 1B). At 30 min after release from mitotic arrest, 20 μM BrdU was added to the medium, together with the indicated concentration of cytochalasin B (Sigma) or (s)-(-)-blebbistatin (Toronto Research Chemicals). Cells were incubated in this medium for 10 h to inhibit cytokinesis, then changed to growth medium containing 20 μM BrdU but no cytokinesis inhibitor, and assayed for S phase entry at the indicated times.

## Authors' contributions

CW participated in the design of the study and carried out the experiments. TS conceived of the study and participated in its design.
